# Long-Term Changes of Subcutaneous Fat Mass in HIV-Infected Children on Antiretroviral Therapy: A Retrospective Analysis of Longitudinal Data from Two Pediatric HIV-Cohorts

**DOI:** 10.1371/journal.pone.0120927

**Published:** 2015-07-06

**Authors:** Sophie Cohen, Steve Innes, Sibyl P. M. Geelen, Jonathan C. K. Wells, Colette Smit, Tom F. W. Wolfs, Berthe L. F. van Eck-Smit, Taco W. Kuijpers, Peter Reiss, Henriette J. Scherpbier, Dasja Pajkrt, Madeleine J. Bunders

**Affiliations:** 1 Department of Paediatric Haematology, Immunology, and Infectious Diseases, Emma Children’s Hospital, Academic Medical Centre, University of Amsterdam, Amsterdam, The Netherlands; 2 KID-CRU (Children's Infectious Diseases Clinical Research Unit), Tygerberg Children’s Hospital and Stellenbosch University, Cape Town, South Africa; 3 Department of Paediatrics, Wilhelmina Children’s Hospital, University Medical Centre Utrecht, University of Utrecht, Utrecht, The Netherlands; 4 Childhood Nutrition Research Centre, University College London, Institute of Child Health, London, United Kingdom; 5 Stichting HIV Monitoring, Amsterdam, The Netherlands; 6 Department of Nuclear Medicine, Academic Medical Centre, University of Amsterdam, Amsterdam, The Netherlands; 7 Department of Global Health and Amsterdam Institute of Global Health and Development, Academic Medical Centre, University of Amsterdam, Amsterdam, The Netherlands; University of Cape Town, SOUTH AFRICA

## Abstract

**Objective:**

Longitudinal studies objectively evaluating changes in regional fat distribution of HIV-infected children assessed by whole body dual energy X-ray absorptiometry (DEXA) are scarce, whilst this long-term effect of HIV and antiretroviral therapy (cART) is an important issue in infected children in need for lifelong treatment.

**Methods:**

We assessed regional fat distribution over time, measured with sequential DEXA-scans in HIV-infected children on cART in cohorts from South Africa (SA) and the Netherlands (NL), and in healthy controls (SA). Limb and trunk fat Z-scores were calculated with the lambda-mu-sigma (LMS) method. Multivariable linear regression models with mixed effects were used to investigate the effect of cART compounds on body fat distribution over time.

**Results:**

In total, 218 children underwent 445 DEXA assessments with a median follow-up of 3.5 years. Fat mass in all limbs was decreased in HIV-infected children compared to controls (arm fat Z-score: coefficient -0.4813; *P* = 0.006, leg fat Z-score: coefficient -0.4345; *P* = 0.013). In the HIV-infected group, stavudine treatment was associated with lower subcutaneous fat mass (arm fat Z-score: coefficient -0.5838; *P* = 0.001), with an additional cumulative exposure effect (arm fat Z-score: coefficient -0.0867; *P* = 0.003).

**Conclusions:**

Our study shows that subcutaneous fat loss is still prevalent in HIV-infected children on cART, and is strongly associated with cumulative stavudine exposure. These results underline the need for early detection of subcutaneous fat loss and alternative treatment options for HIV-infected children globally.

## Introduction

The scale-up of combination antiretroviral therapy (cART) has resulted in a rapidly growing number of HIV-infected patients receiving cART globally. In view of the need for lifelong treatment, the impact of several short- and long-term complications of cART has become increasingly important, especially for HIV-infected children [1]. Changes in fat metabolism and distribution are amongst the most important of these long-term complications [2,3]. These changes are physically manifested as lipoatrophy (loss of subcutaneous fat) and lipohypertrophy (visceral fat accumulation) [4]. Lipoatrophy is associated with stigma and reduced therapy adherence, in particular in children and young adolescents [5]. The accumulation of visceral fat affects metabolic and inflammatory processes and is consequently associated with a higher risk of coronary artery disease and diabetes mellitus type II [3,6]. Although the underlying mechanisms may differ, lipoatrophy and lipohypertrophy can occur simultaneously.

Specific antiretroviral compounds, in particular the nucleoside reverse transcriptase inhibitors (NRTIs) have been implicated in the aetiology of lipoatrophy [7–10]. NRTIs, especially stavudine and zidovudine, inhibit mitochondrial DNA polymerase gamma activity and subsequent mitochondrial functioning, resulting in a decrease in lipogenesis and an increase in lipoapoptotic mediators [11,12]. Until 2010, the World Health Organization’s (WHO) first-line regimen options for HIV-infected children included both stavudine and zidovudine. Although WHO guidelines no longer recommend it, many children in sub-Saharan Africa continue to receive stavudine as part of their cART regimen [13], as is the case for zidovudine. Other components of cART, such as protease inhibitors (PIs) are also reported to have an effect on regional fat distribution and fat metabolism [4,10,14]. Recently, elevations in low density lipoprotein and triglycerides in children on a lopinavir/ritonavir (lopinavir/r) based cART regimen were reported, as well as changes in body fat composition [10]. With the latest WHO guidelines recommending lopinavir/r as firstline treatment for children under three years of age [1], these findings require further assessment.

Assessing regional fat mass accurately and objectively is challenging. Pediatric studies have predominantly used visual assessment, anthropometry and bioelectrical impedance with a high variability [7–10,15]. Dual Energy X-ray Absorptiometry (DEXA) has proved to be a reliable method providing consistent and detailed information on regional fat mass. Recently, body composition of a cohort of HIV-infected children on cART was assessed in a study on the prevalence of visually obvious lipoatrophy in Cape Town, South Africa [9]. A subset of children in this cohort also underwent DEXA. In the Netherlands, bone mineral density and regional body fat of HIV-infected children on cART has been monitored by DEXA for clinical purposes since 2002 in the Academic Medical Centre in Amsterdam and the Utrecht University Medical Centre. Together, these two cohorts provide the unique opportunity to assess changes over time in regional fat mass in cART-treated, HIV-infected children on two continents.

## Methods

### Ethics Statement

In the Netherlands, all DEXA scans were obtained for clinical purposes and results were collected and analysed anonymously. The demographic, HIV- and cART-related information was obtained from the HIV monitoring foundation database. The HIV monitoring foundation database includes anonymized data from all HIV-infected children living in the Netherlands who receive care in one of the four pediatric HIV treatment centers. HIV-infected children and their caregivers are informed about the data collection by their treating physician and patients can object to further collection according to an opt-out procedure. Written informed consent and ethical approval is not obtained, as data collection is part of HIV care in the Netherlands.

For the South African cohort the Ethics Committee for Human Research of the Stellenbosch University approved the study. Written informed consent was obtained from each caregiver and informed consent was obtained from capable children.

All patient-related data were stored in a secured database under a patient identifying number and kept strictly confidential.

### Participants

Participants were included from 2 cohorts of HIV-infected children: 1) from the Netherlands in care at the Academic Medical Centre (Amsterdam) and University Medical Centre (Utrecht); and 2) from Tygerberg Children’s Hospital in South Africa (Cape Town). In the South African cohort, age-, gender-, and socioeconomically-matched healthy controls from the same community as the HIV-infected children were also included [16]. Of note, most children from the Netherlands are black or mixed black, and are first or second-generation immigrants from sub-Saharan African descent [17].

In the Netherlands, all HIV-infected children on cART underwent consecutive whole body DEXA-scans with median intervals of 1.9 years (IQR 1.5 to 2.7) to monitor bone mineral density and regional fat distribution for clinical purposes [18]. All DEXA-scans performed from January 2002 until May 2012 were included in this study.

In South Africa, cART-treated, pre-pubertal children between 3–12 years old were recruited between February 2010 and January 2011 for an earlier study on lipoatrophy as quantified by skin fold thickness [9]. In this study, 100 children were included (190 subjects met the inclusion criteria, 121 agreed to participate yet 21 did not attend the study visits). There were no demographic differences between the enrolled and unenrolled subjects (*P*-value>0.20 for age, gender, cumulative time on stavudine and CD4^+^ T-cell count). In addition to skin fold measurements, as many study participants as logistically possible underwent DEXA scans (n = 77), which were the participants included in the current study. There was no difference in gender, cumulative time on stavudine or CD4^+^ T-cell count between subjects who underwent DEXA and those who did not (*P*-value>0.50 for all). After the initial referral to the tertiary hospital upon diagnosis and inclusion in the study, a proportion of children were transferred to local clinics and not under care after 1 year. Therefore, only 32 (42%) of the 77 children underwent a follow-up DEXA scan. The children with a second DEXA scan had a median 1.2 year interval between both scans (IQR 1.1 to 1.3).

### Study parameters

For the Dutch cohort, demographic and HIV/cART-related parameters, and Centre for Disease Control and prevention (CDC) classifications were extracted from the Dutch HIV Monitoring Foundation database [19]. In South Africa this information was derived from the electronic health record database and the central electronic laboratory results server [9]. The CDC classifications from the Netherlands were manually changed to WHO disease stages. Ethnicity was divided into four main groups; black, white, mixed black (children from the Dutch cohort with one black and one white parent), and mixed ethnicity. Mixed ethnicity was defined as a heterogeneous ethnic group living in Cape Town, with ancestry from Europe, Malaysia and Southern Africa.

### DEXA scans

In the Netherlands, scans were performed on the Hologic DEXA scanner (QDR4500W; Hologic Inc, Waltham, MA), on which trunk and individual limb fat mass (grams), lean mass (grams) and fat percentage were determined. In September 2011, the Hologic QDR4500W scanner in the Netherlands was replaced by the Hologic Discovery, calibrated to the previous machine with no effect on the output generated. In South Africa, a Hologic Discovery was used for all scans. All scans were performed and processed according to the same manufacturer’s protocol. DEXA-output from both centres was exchanged and re-evaluated by investigators from the other centre, providing similar results.

### Statistical Analysis

All statistical analyses were carried out using Stata IC version 10, 2009 (StataCorp, Texas). Descriptive statistics were performed on the demographic and HIV-related characteristics. Designated tests for parametric (Student’s T-test) and non-parametric (Kruskal-Wallis and Mann-Whitney U) numerical data, and the Chi-square test for categorical data were used to compare variables between groups.

A DEXA scan assesses fat, bone and lean body mass of the arms, legs and trunk, providing separate measurements for these body compartments. We analysed the left leg, left arm, trunk fat and left arm fat versus lean ratio as these have shown consistent results in previous studies on lipoatrophy and lipohypertrophy [20].

Instead of assessing absolute measurements, we used age-adjusted Z-scores in our analyses. Age-adjusted Z-scores provide the opportunity to analyse body fat measurements over time, as a child’s regional fat mass varies substantially with age. The absolute measures were transformed using 2 methods: 1) Age related Z-scores were constructed using all study participants (including the healthy controls) as the standard, providing the opportunity to evaluate how an individual participants’ measurement compares in Z-score to the rest of the study group. This is a robust method to generate Z-scores for this group as it includes the largest number of measurements. Secondly, it provides a measure of reference when comparing parameters of fat mass distribution in HIV-infected children from South Africa and the Netherlands. 2) Recently, a standard of regional fat mass of children in the United Kingdom was published and we used this standard to create a second set of age-related Z-scores of the absolute measurements of our study participants [21]. This latter method provides the opportunity to control for potential differences between regions. In both cases the age-adjusted Z-scores were obtained using the lambda-mu-sigma (LMS) method. The LMS method enables calculation of normalized centile standards and transforms measurements into Z-scores, as previously described [22]. The arm fat versus lean ratio was not transformed into Z-scores as it varies less with age [20,23]. So although the aim of the study was to investigate HIV- and cART-related parameters in HIV-infected children, we aimed to identify the magnitude of the differences compared to a normal population [9,24,25] by 1. comparing the HIV-infected children with uninfected children from South Africa and 2. deriving Z-scores from a European standard.

Within the HIV-infected group, the effects of various demographic, HIV- and cART-related variables on body fat distribution over time were assessed with multivariable linear regression models on the LMS-calculated Z-scores. Mixed effects linear regression models incorporating maximum likelihood estimation were generated to take into account repeated measurements and changes over time within individuals. In these models, the number of DEXA scans was used as the panel variable, and the age at DEXA scan as time variable. Therefore, the coefficient generated with mixed effects linear regression models describes an effect ‘over time’. All models were adjusted for region of origin to account for differences between South African children and children living in the Netherlands. To assess whether there was a duration effect of treatment with a specific compound, we repeated the analyses substituting the significantly associated cART compound with a time dependent variable. A variable with a coefficient with a *P*-value <0.2 in univariable analysis was included in the multivariable model.

A sub-analysis was performed on children who ceased stavudine treatment after their first DEXA and had one or more subsequent DEXA scans after switching away from stavudine. A Wilcoxon-signed-rank-test was used to assess the changes over time in the arm and leg Z-scores in this subgroup of HIV-infected children.

## Results

### Demographic data

A total of 218 children were included in the study; 98 HIV-infected from the Netherlands (NL), 77 HIV-infected-, and 43 uninfected healthy children from South Africa (SA) (*Table 1*). At their first DEXA scan children from the Netherlands were older (median 7·4 years, IQR 5.1 to 10.2) than the South African group (median for HIV-infected children 6.3 years, IQR 4.9 to 8.6; *P*<0.01). The majority of both groups was black (NL: 68%, SA: 52%) or had a mixed ethnicity (NL: 20%, SA: 48%). Despite a higher length- and weight-for-age in the HIV-infected children from the Dutch cohort, there was no difference in median BMI-for-age between HIV-infected children from both countries (NL: 0.4 (IQR -0.5 to 1.1); SA: 0.4 (IQR -0.4 to 1.1)) (Table 1).

**Table 1:** Participant characteristics at first DEXA scan.

		HIV-infected children				Healthy children	
**Demographic variables**		**n**	**NL (n = 98)**	**n**	**SA (n = 77)**	**n**	**SA (n = 43)**
Gender	Female	98	51 (52)	77	34 (44)	43	20 (47)
	Male		47 (48)		43 (56)		23 (53)
Year of birth	<1996	98	35 (36)	77	0 (0)	43	0 (0)
	1996–2003		58 (59)		18 (23)		2 (5)
	>2003		5 (5)		59 (77)		41 (95)
Age (years)		98	7.4 (5.1 to 10.2)	77	6.3 (4.9 to 8.6) **	43	5.2 (5.0 to 5.7) ^**◊◊**^
Ethnicity	Black	91	62 (68)	76	41 (54)	43	21 (49)
	White		11 (12)		0 (0)		0 (0)
	Mixed ethnicity		0 (0)		35 (46)		22 (51)
	Mixed black		19 (20)		0 (0)		0 (0)
Height for age (Z)		98	-0.1 (-1.0 to 0.7)	75	-1.3 (-1.9 to -0.9) **	43	-1.1 (-1.7 to -0.5)
Weight for age (Z)		72	0.3 (-0.6 to 1)	68	-0.5 (-1.2 to 0.1) **	42	0·0 (-1.0 to 0.8) ^**◊**^
BMI for age (Z)		98	0.4 (-0.5 to 1.1)	75	0.4 (-0.4 to 1.1)	43	0.6 (0.0–1.4)
Duration follow-up (y)		75	4.8 (2.8 to 6.7)	32	1.2 (1.2 to 1.3)	28	1.2 (1.1–1.3)
Number of DEXA scans		98	2.7 (1to 6)	77	1.4 (1 to 2)	43	1.7 (1–2)
**HIV-associated variables**							
Maximum WHO clinical stage	0–2	78	24 (31)	77	7 (9)		NA
	3		8 (10)		33 (43)		NA
	4		46 (59)		36 (47)		NA
HIV VL (log cop/mL) at first DEXA		97	1.7 (1.7 to 2.5)	74	1.8 (1.6 to 2.5)		NA
Undetectable HIV VL at first DEXA	>500 cop/mL	97	22 (23)	74	8 (11)		NA
	<500 cop/mL		75 (77)		66 (89)		NA
CD4+ T-cell count (*10^6^/L)	cART initiation	90	441 (194 to 1050)	35	753 (375 to 1089)		NA
	first DEXA	95	930 (620 to 1260)	74	1142·5 (822 to 1524) **		NA
CD4+ T-cell count (%)	cART initiation	87	16 (8 to 26)	36	17 (13 to 23)		NA
	first DEXA	92	33 (25 to 37)	74	32 (27 to 38)		NA
Duration cART at first DEXA (y)		97	3.1 (1.3 to 5.0)	76	4.4 (3.1 to 6.4)		NA
Total exposure time to cART (y)		97	7.6 (3.9 to 10.5)	76	5.1 (3.6 to 7.1)		NA
Age at cART initiation (y)		97	3.5 (1.2 to 6.6)	77	1.8 (0.7 to 3.2) **		NA
Pre-treated with mono therapy		97	17 (17)	76	0 (0)		NA
cART compounds at first DEXA	Abacavir		38 (39)		33 (43)		NA
	Stavudine		52 (53)		67 (87)		NA
	Tenofovir		7 (7)		0 (0)		NA
	Zidovudine		47 (48)		19 (25)		NA
	Lopinavir		29 (30)		55 (71)		NA
	Nelfinavir		44 (45)		0 (0)		NA
	Efavirenz		46 (47)		26 (33)		NA
	Nevirapine		15 (15)		0 (0)		NA

DEXA = dual X-ray absorptiometry. SA = South Africa. NL = the Netherlands. Z = Z-score. BMI = Body Mass Index. Y = years. WHO = World Health Organisation. NA = not applicable. HIV VL = HIV viral load. (Log) cop/mL = (log) copies/mL. cART = combination antiretroviral therapy. Values are n (%) or median (IQR).

** = *P*<0.01 (NL HIV+ vs. SA HIV+)·

^◊^ = *P*<0.05 (SA HIV+ vs. SA HIV-)

^◊◊^ = *P*<0·01 (SA HIV+ vs. SA HIV-). All children were exposed to lamivudine at first DEXA.

In South Africa 90% of the children had a pre-cART WHO clinical stage 3 or 4 at baseline, compared to 69% of children from the Netherlands (Table 1), with no difference in HIV viral load and CD4^+^ T-cell percentage. CART exposure time was similar between children from the Netherlands and South Africa. At first DEXA scan, a larger proportion of children in South Africa were treated with stavudine (NL: n = 52, 53%, SA: n = 67, 87%) and lopinavir (NL: n = 29, 30%, SA: n = 55, 71%) compared to Dutch children, while a higher percentage of Dutch children was treated with zidovudine (NL: n = 47, 48%, SA: n = 19, 25%) and efavirenz (NL: n = 46, 47%, SA: n = 26, 33%).

### Longitudinal data

The study included 445 DEXA scans, including 373 scans from HIV-infected children. The median duration of follow-up of the children in the Netherlands with more than 1 DEXA scan (n = 75) was 4.8 years (IQR 2.8 to 6.7), with a maximum of 9.4 years. The South African cohort included 109 scans of HIV-infected children with 32 children having 2 consecutive scans with a median follow-up of 1.2 years (IQR 1.2 to 1.3, range 0.4 to 2.2). South African children with and without a follow-up scan were similar in gender (*P*-value = 0.631), age at ART initiation (*P*-value = 0.562), CD4^+^ T-cell percentage (*P*-value = 0.167), HIV viral load (*P*-value = 0.614), ART exposure years (*P*-value = 0.412), cumulative stavudine exposure (*P*-value = 0.980) and cumulative lopinavir exposure (*P*-value = 0.686) (all at first DEXA scan). In the healthy controls 71 scans were performed with 28 children having 2 consecutive scans with a median follow-up of 1.2 years (IQR 1.1 to 1.3, range 0.4 to 1.6).

We confirmed with multivariable regression analyses adjusting for country of origin, gender, and ethnicity that the HIV-infected children had a lower left arm fat Z-score (coefficient -0.4813, *P* = 0.006), left leg fat Z-score (coefficient -0.4345, *P* = 0.013) and arm fat versus arm lean ratio (coefficient -0.1295, *P* = 0.010) compared to the healthy group from South Africa (Data not shown). Importantly, no significant association was found between country of origin and limb fat Z-scores (arm: coefficient -0.2449, *P* = 0.159; leg: coefficient 0.1573, *P* = 0.366). Moreover, female gender was positively associated with both limb and trunk fat mass in the multivariable analyses (arm: coefficient 0.3641, *P-*value = 0.004; leg: coefficient 0.6707, *P-*value<0.001; trunk: 0.5071, *P-*value<0.001).

We established that by using only the data of the South African HIV-infected and uninfected children, the HIV-associated differences remained (left arm Z-score: coefficient -0.4678, *P* = 0.009; leg: coefficient -0.4078, *P* = 0.027). Furthermore, these analyses were repeated using the Z-scores created with the UK reference data [21]. HIV-infected children had a -0.6 (IQR: -1.3 to 0) arm fat mass Z-score and a -1.7 (IQR: -2.4 to -1.0) leg fat mass Z-score at their first DEXA scan, while the uninfected participants had an arm fat mass Z-score of 0 (IQR -0.6 to 0.4) and a leg fat mass Z-score of -0.9 (IQR -1.4 to -0.3) compared to the UK standard. Multivariable regression analyses using these Z-scores again showed lower limb fat Z-scores over time in the HIV-infected group compared to the healthy children (arm: coefficient -0.4404, *P* = 0.015; leg: coefficient -0.6141, *P* = 0.002).

### Effect of antiretroviral treatment on regional fat mass in HIV-infected children

**Fig 1:**
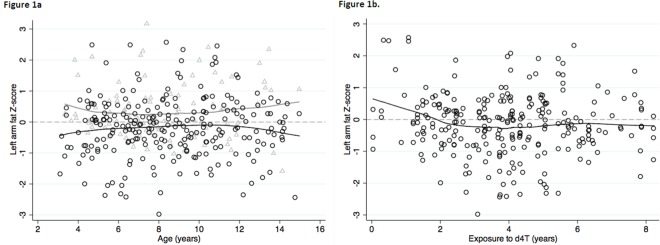
a. Left arm fat Z-scores of HIV-infected children over age. Black circles: scatterplot and locally weighted scatterplot smoothing line (LOWESS) of children exposed to stavudine; Grey triangles: scatterplot and LOWESS of children not exposed to stavudine. b. Left arm fat Z-scores of HIV-infected children over years of stavudine exposure. Black circles: scatterplot and locally weighted scatterplot smoothing line (LOWESS) of children exposed to stavudine.

To evaluate the underlying factors explaining the loss of subcutaneous fat in HIV-infected children in these cohorts, we assessed associations between HIV-related parameters and specific antiretroviral compounds with regional fat mass over time. Univariable regression analyses of the left arm fat Z-score showed *P-*values <0.2 for gender, ethnicity, region of origin, CD4^+^ T-cell count, maximum WHO clinical stage, stavudine and lopinavir/r treatment. These variables were included in the multivariable model (Table 2). In the multivariable model, treatment with stavudine remained significantly associated with a lower left arm fat Z-score over time (coefficient -0.5838, *P* = 0.001) (Fig 1A) and there was a trend towards lower left arm Z-score over time in children treated with lopinavir/r (coefficient -0.2177, *P* = 0.099) (Table 2). Treatment with stavudine was also negatively associated with the arm fat versus lean ratio (coefficient -0.1670, *P* = 0.001). Lopinavir/r showed no association with this ratio (coefficient 0.0087, *P* = 0.778) (S1 Table). We further assessed whether a time-dependent effect of stavudine and lopinavir/r was present, by repeating the uni- and multivariable analyses and substituting the cART-exposure with the cumulative treatment duration of these antiretroviral compounds. Duration of treatment with stavudine was associated with a lower left arm fat Z-score over time (coefficient -0.0867, *P* = 0.003) as well as with the arm fat to lean ratio (coefficient -0·0294, *P*<0·001) (Table 2 and S1 Table). The left arm fat Z-score of the stavudine-treated HIV-infected children most markedly decreased in the first 2 years of exposure and then stabilized (Fig 1B). Longer duration of treatment with stavudine was borderline significantly associated with a decreasing leg fat Z-score (coefficient -0.0502, *P* = 0.051) (*Table 3*). In univariable analysis, a longer duration of treatment with lopinavir/r was associated with a lower left arm fat Z-score over time (coefficient -0.0781, *P* = 0.001), however when assessed in multivariable analysis the coefficient was -0.0403 with a *P*-value of 0.127 (Table 2).

**Table 2:** Univariable and multivariable analyses of left arm fat Z-scores in HIV-infected children.

			Left arm fat Z-Score			
			Univariable Analysis		Multivariable Analysis	
Characteristics		n	Coefficient	*P*-value	Coefficient	*P*-value
HIV VL at DXA scan	<500	140	-	-	-	-
	30		0.0975	0.469	-	-
Absolute CD4 count at DEXA		168	-0.0002	0.028^**◊**^	-0.0002	0.094
Maximum WHO clinical stage	0–2	31	-	-	-	-
	3	40	-03965	0.072^**◊**^	-0.0653	0.780
	4	82	-0.1645	0.392	-0.0080	0.967
*Treatment*						
Abacavir		106	-0.0028	0.976	-	-
Stavudine		119	-0.5063	0.001^**◊**^	-0.5838	0.001*
Tenofovir		22	0.1417	0.343	-	-
Zidovudine		73	0.1604	0.208	-	-
Lopinavir		94	-0.3285	0.006^**◊**^	-0.2177	0.099
Nelfinavir		44	0.1128	0.473	-	-
Efavirenz		104	-0.0104	0.912	-	-
*Treatment duration (years)*						
Absolute CD4 count at DEXA		168	-0.0002	0.028^**◊**^	-0.0002	0.049*
Maximum WHO clinical stage	0–2	31	-	-	-	-
	3	40	-0.3965	0·072^**◊**^	-0.1540	0.509
	4	82	-0.1645	0.392	-0.0980	0.608
Stavudine		119	-0.0908	0.001^**◊**^	-0.0867	0.003*
Lopinavir		94	-0.0781	0.001^**◊**^	-0.0403	0.127

HIV VL = HIV viral load. DEXA = dual energy X-ray absorptiometry. WHO = World Health Organization. Multivariable analyses are adjusted for gender, ethnicity and country of origin. Lamivudine was used in all children and was therefore not included in the models.

^◊^ = P<0.2 in univariable analysis

* = P<0.05 after multivariable analysis

**Table 3:** Univariable and multivariable analyses of left leg fat Z-scores in HIV-infected children.

			Left leg fat Z-scores			
			Univariable Analysis		Multivariable Analysis	
HIV- and cART characteristics		n	Coefficient	*P*-value	Coefficient	*P*-value
HIV VL at DEXA	<500	140	-	-	-	-
	>500	30	-0.1031	0.387	-	-
Absolute CD4^+^ T-cell count at DEXA		168	-0.0003	0.001^◊^	-0.0003	0.001*
Maximum WHO clinical stage	0–2	31	-	-	-	-
	3	40	-0.0494	0.831	-	-
	4	82	-0.1214	0.548	-	-
*Treatment*						
Abacavir		106	0.1550	0.064^◊^	0.1455	0.077
Stavudine		119	-0.2404	0.125^◊^	-0.1322	0.398
Tenofovir		22	0.1951	0.139^◊^	0.0407	0.767
Zidovudine		73	0.1076	0.387	-	-
Lopinavir		94	0.0049	0.966	-	-
Nelfinavir		44	-0.0525	0.751	-	-
Efavirenz		104	0.0612	0.467	-	-
*Treatment duration (years)*						
Absolute CD4^+^ T-cell count at DEXA		168	-0.0003	0.001^◊^	-0.0002	0.007*
Abacavir		106	0.0847	<0.001^◊^	0.0738	<0.001*
Stavudine		119	-0.0542	0.042^◊^	-0.0502	0.051
Tenofovir		22	0.0525	0.194^◊^	0.0046	0.693

HIV VL = HIV viral load. DEXA = dual energy X-ray absorptiometry. WHO = World Health Organization. Multivariable analyses are adjusted for gender, ethnicity and country of origin. Lamivudine was used in all children and was therefore not included in the models.

^◊^ = P<0.2 in univariable analysis

* = P<0.05 after multivariable analysis

In a sub-analysis we assessed whether treatment with stavudine and lopinavir/r together lead to an enhanced decrease in subcutaneous fat mass compared to treatment with stavudine alone. The multivariable linear regression analysis adjusting for gender, ethnicity, region of origin, absolute CD4 count and maximum WHO clinical stage showed no significant add-on effect of exposure to both cART compounds compared to stavudine alone (arm fat: coefficient -0.0475, *P* = 0.838; leg fat: coefficient -0.0099, *P* = 0.966).

Several cART compounds were positively associated with the DEXA-derived limb fat parameters. Cumulative treatment with abacavir was positively associated with left leg fat Z-score over time (coefficient 0.0738, *P*<0.001) (Table 3). Prolonged exposure time to tenofovir was positively associated with the arm fat versus lean ratio (coefficient 0.0332, *P* = 0.001) and the trunk fat Z-score over time (arm fat versus lean ratio: coefficient 0.0332, *P* = 0.001, trunk fat: coefficient 0.1131, *P* = 0.026) (S1 and S2 Tables).

To support our findings showing associations with cART compounds and changes of subcutaneous fat mass over time, we repeated the regression analyses described above using Z-scores based on the UK norm, detecting similar associations regarding HIV and cART related factors. To illustrate, we detected equivalent negative associations between exposure to stavudine and lopinavir/r with left arm fat over time (stavudine: coefficient -0.5064, *P* = 0.007; lopinavir/r: coefficient -0.2826, *P* = 0.039). The associations with cumulative exposure and arm fat mass over time were similar too (stavudine: coefficient -0.1131, *P*<0.001; lopinavir/r: coefficient -0.0530, *P* = 0.051). Interestingly, in this analysis which uses gender specific Z-scores, we observed a negative association with female gender and fat mass, (left arm fat Z-score: coefficient -0.3813, *P*-value 0.006; trunk fat Z-score: coefficient -0.4397, *P*-value 0.001; left leg fat Z-score: coefficient -0.3281, *P*-value 0.039). This finding implies that although girls may still have a larger absolute fat mass, which the earlier analyses showed, the decrease is larger in girls than in boys.

Reversibility of fat loss after ceasing of stavudine treatment is still debated [25,26]. In our cohort a subgroup of 26 children switched from stavudine to another NRTI (predominantly abacavir) and had DEXA assessments before as well as after this switch. The majority of these children resided in the Netherlands (N = 21, 81%). They had been treated with stavudine for a median of 4.5 years (IQR 3.5 to 5.8) until switching, and with an alternative NRTI for a median of 3.0 years at their final fat mass (DEXA) assessment (IQR 1.4 to 5.7). Left arm fat Z-score did not increase after the switch; based on the UK reference data, their median left arm fat Z-score was -0.4 (IQR -1.0 to 0.2) before the switch and -0.9 (IQR -1.6 to 0.0) at the last DEXA-scan after the switch (*P* = 0.16). Their median left leg fat Z-score was -1.6 (IQR -2.7 to -0.7) before the switch and -1.8 (IQR -3.1 to -1.0) after the switch (*P* = 0.11).

## Discussion

Using longitudinal data of two pediatric cohorts from South Africa and the Netherlands, we demonstrate that treatment with stavudine is strongly associated with a reduced limb fat mass in a cumulative, time-dependent manner. This finding is consistent with prior studies in both HIV-infected adults and children [4,7,9,10,13,23]. In a small subset of patients in our study, there was no evidence of reversibility of subcutaneous fat loss after replacing stavudine. Previously, pediatric studies showed that switching away from stavudine may lead to recovery of lipoatrophy [25–27]. In longitudinal studies in HIV-infected adults, partial recovery of fat mass has also been observed, however patients on stavudine were least likely to fully recover [28,29]. Treatment with stavudine is no longer recommended in the WHO guidelines for the treatment of HIV-infection in children [1], however it is still widely used in areas where alternatives are sparsely available. Our findings emphasize the need to screen for lipoatrophy when treatment with stavudine is unavoidable.

Apart from the strong association of stavudine treatment and the decrease of subcutaneous fat over time, we detected a trend towards a reduced subcutaneous fat mass in children treated with lopinavir/r, which is currently advised by WHO guidelines as first line regimen in children under three years of age [1]. PIs in general and lopinavir/r in particular have been mentioned as a cause for changes in fat metabolism and distribution, albeit to a lesser extent than stavudine [7,10]. Our data showed a trend towards an association between lopinavir/r and subcutaneous fat loss. This could be due to the smaller numbers included in our study and caution regarding children on lopinavir/r may still be warranted.

Abacavir and tenofovir were positively associated with regional fat mass in our study and may provide alternatives for stavudine. Moreover, zidovudine showed no negative association with subcutaneous fat mass and may also be considered. However, the anemia-inducing effect of zidovudine limits its usefulness, especially in sub-Saharan Africa, and as in adults on zidovudine, it may just take longer as compared to stavudine for lipoatrophy to develop [30]. Abacavir, which in fact had a positive association with the leg fat mass in our study, may have a reduced long-term efficacy in HIV-treated infants [31]. Lastly, tenofovir, firstline treatment in adults, is avoided in children due to concerns about its renal and bone toxicities [18,32]. This indicates that although stavudine induces lipoatrophy, appropriate alternatives are limited.

Of note, HIV infection itself also affects adipose tissue [33], and effective treatment of HIV is essential to halt HIV-associated loss of fat mass in children. In the combined cohorts as well as using the South African HIV-infected group only, we confirmed that HIV-infected children had a lower limb fat mass compared to healthy children. This difference between HIV-infected and uninfected children is likely to be not completely drug-induced, and the importance of effective treatment of HIV to prevent an abnormal fat mass distribution due to the infection should therefore not be disregarded.

DEXA is a precise method for body composition assessment [34]. However, instruments of different manufacturers give different values for soft tissue. The Hologic QDR4500W tends to give higher values for fat mass than the Lunar instrumentation, which was used for the UK reference data [35]. This means that loss of fat mass in HIV-infected children compared to healthy children from Western Europe is likely to be larger than presented in this study. We decided to present the regression analyses derived from Z-scores created within our own study group, as they were all measured with the same instruments and more homogeneous. Of note, investigators from both centres re-evaluated and confirmed the DEXA results from the other centre, however due to the retrospective nature of the study the DEXA-scanners were not phantom-calibrated before the start of data collection. Thus, the retrospective design of this study is a major limitation, including the lack of healthy controls from the Netherlands. Nonetheless, the main study question concerned risk factors for lipodystrophy in HIV-infected children on cART, which confirmed a detrimental effect of stavudine. The combination of the two cohorts with different follow-up protocols was accounted for using mixed linear regression models adjusted for country of residence and the varying numbers of DEXA scans per individual. Of note, 45 South African children (58%) were lost-to-follow-up. However, they did not differ in demographic-, HIV- or ART-related characteristics, and they were transferred to local clinics due to logistic reasons rather than patient or disease-related characteristics. Therefore, the lack of follow-up scans from a significant proportion of the South African cohort was unlikely to have resulted in selection bias. Length- and weight for age was different between groups from both centres, however despite environmental and dietary differences between both groups, their BMI was similar. BMI has previously been shown to correlate strongly with body fat distribution and for the purpose of body fat distribution analysis over time it was the most important parameter to match between groups [36,37]. We were unable to analyse lipohypertrophy because DEXA cannot distinguish between visceral and subcutaneous abdominal fat, and only provides an indication of trunk fat. As a result, co-existence of lipoatrophy and lipohypertrophy may have been indistinguishable from normal abdominal fat distribution. Alternatives such as MRI are required to detect associations between antiretroviral compounds and changes in visceral fat. Lastly, we attempted to find evidence on reversibility of lipoatrophy, an important topic that is still debated. However, the subgroup that switched away from stavudine in our study was likely to be too small to reveal potential restoration of subcutaneous fat mass.

In conclusion, this study shows an objectively measured decrease of subcutaneous fat mass over time in stavudine-treated, HIV-infected children from South Africa and the Netherlands. Furthermore, the use of protease inhibitors needs to be further investigated in the context of changes in regional fat mass. The ongoing use of stavudine in sub-Saharan Africa and the potentially irreversible nature of peripheral lipoatrophy underline the need for early detection of changes in subcutaneous fat and alternative treatment options for HIV-infected children globally.

## Supporting Information

S1 Table Univariable and multivariable analyses of the arm fat to arm lean ratio in HIV-infected children. HIV VL = HIV viral load. DEXA = Dual Energy X-ray Absorptiometry. WHO = World Health Organisation. Multivariable analyses are adjusted for gender and country of origin. Lamivudine was used in all children and was therefore not included in the models. ^◊^ = P<0.2 in univariable analysis. * = P<0.05 after multivariable analysis.
(DOC)

S2 Table Univariable and multivariable analyses of trunk fat Z-scores in HIV-infected children. HIV VL = HIV viral load. DEXA = Dual Energy X-ray Absorptiometry. WHO = World Health Organisation. Multivariable analyses are adjusted for gender and country of origin. Lamivudine was used in all children and was therefore not included in the models. ^◊^ = P<0.2 in univariable analysis. * = P<0.05 after multivariable analysis.
(DOC)
