# Correction: Long-Term Changes of Subcutaneous Fat Mass in HIV-Infected Children on Antiretroviral Therapy: A Retrospective Analysis of Longitudinal Data from Two Pediatric HIV-Cohorts

**DOI:** 10.1371/journal.pone.0190726

**Published:** 2018-01-02

**Authors:** Sophie Cohen, Steve Innes, Sibyl P. M. Geelen, Jonathan C. K. Wells, Colette Smit, Tom F. W. Wolfs, Berthe L. F. van Eck-Smit, Taco W. Kuijpers, Peter Reiss, Henriette J. Scherpbier, Dasja Pajkrt, Madeleine J. Bunders

There is missing information in the Funding section. The correct funding information is as follows: SI received a pilot research grant (P30 AI036214-16, sub-award 10304442) from the University of California San Diego Centre for AIDS Research (CFAR), a Fogarty International Clinical Research Fellowship grant (R24-TW007988-01), a Southern Africa Consortium for Research Excellence grant (WTX055734) from the Wellcome Trust, and a research grant from Collaborative Initiative for Paediatric HIV Education and Research (CIPHER; 158-INN). The funders had no role in study design, data collection and analysis, decision to publish, or preparation of the manuscript.
