# Supplementary material for: Long-Term Changes of Subcutaneous Fat Mass in HIV-Infected Children on Antiretroviral Therapy: A Retrospective Analysis of Longitudinal Data from Two Pediatric HIV-Cohorts
Source: PLoS One. 2015 Jul 6;10(7):e0120927. doi: 10.1371/journal.pone.0120927 (PMC4493065; doi:10.1371/journal.pone.0120927)
Supplement: S1 Table — HIV VL = HIV viral load. DEXA = Dual Energy X-ray Absorptiometry. WHO = World Health Organisation. Multivariable analyses are adjusted for gender and country of origin. Lamivudine was used in all children and was therefore not included in the models. ◊ = P<0.2 in univariable analysis. * = P<0.05 after multivariable analysis. (DOC) [file pone.0120927.s001.doc]

**Supplementary table 1. Univariable and multivariable analyses of the arm fat to arm lean ratio in HIV-infected children**

|  |  |  | **Arm fat: Arm lean ratio** | | | |
| --- | --- | --- | --- | --- | --- | --- |
|  |  |  | **Univariable Analysis** | | **Multivariable Analysis** | |
| **HIV- and cART characteristics** |  | **n** | **Coefficient** | ***P*-value** | **Coefficient** | ***P*-value** |
| HIV VL at DEXA scan | <500 | 140 | - | - | - | - |
|  | >500 | 30 | -0.0021 | 0.943 | - | - |
| Absolute CD4^+^ T-cell count at DEXA |  | 168 | <0.0001 | 0.300 | - | - |
| Maximum WHO clinical stage | 0-2 | 31 | - | - | - | - |
|  | 3 | 40 | 0.0490 | 0.467 | - | - |
|  | 4 | 82 | 0.0422 | 0.474 | - | - |
| *Treatment* |  |  |  |  |  |  |
| Abacavir |  | 106 | -0.0026 | 0.904 | - | - |
| Stavudine |  | 119 | -0.0894 | 0.042^◊^ | -0.1670 | 0.001* |
| Tenofovir |  | 22 | 0.0543 | 0.099^◊^ | 0.0588 | 0.072 |
| Zidovudine |  | 73 | -0.0129 | 0.699 | - | - |
| Lopinavir |  | 94 | 0.0087 | 0.778 | - | - |
| Nelfinavir |  | 44 | -0.0921 | 0.047^◊^ | 0.0415 | 0.488 |
| Efavirenz |  | 104 | -0.0160 | 0.452 | - | - |
| *Treatment duration (years)* |  |  |  |  |  |  |
| Stavudine |  | 119 | -0.0241 | 0.001^◊^ | -0.0294 | <0.001* |
| Tenofovir |  | 22 | 0.0311 | 0.001^◊^ | 0.0332 | 0.001* |
| Nelfinavir |  | 44 | 0.0023 | 0.389 | 0.0043 | 0.081 |

HIV VL= HIV viral load. DEXA= Dual Energy X-ray Absorptiometry. WHO= World Health Organisation. Multivariable analyses are adjusted for gender and country of origin. Lamivudine was used in all children and was therefore not included in the models.
^◊^=P<0.2 in univariable analysis.
*=P<0.05 after multivariable analysis.
