# Supplementary material for: Long-Term Changes of Subcutaneous Fat Mass in HIV-Infected Children on Antiretroviral Therapy: A Retrospective Analysis of Longitudinal Data from Two Pediatric HIV-Cohorts
Source: PLoS One. 2015 Jul 6;10(7):e0120927. doi: 10.1371/journal.pone.0120927 (PMC4493065; doi:10.1371/journal.pone.0120927)
Supplement: S2 Table — HIV VL = HIV viral load. DEXA = Dual Energy X-ray Absorptiometry. WHO = World Health Organisation. Multivariable analyses are adjusted for gender and country of origin. Lamivudine was used in all children and was therefore not included in the models. ◊ = P<0.2 in univariable analysis. * = P<0.05 after multivariable analysis. (DOC) [file pone.0120927.s002.doc]

**Supplementary table 2. Univariable and multivariable analyses of trunk fat Z-scores in HIV-infected children**

|  |  |  | **Trunk fat Z-scores** | | | |
| --- | --- | --- | --- | --- | --- | --- |
|  |  |  | **Univariable Analysis** | | **Multivariable Analysis** | |
| **HIV- and cART characteristics** |  | **n** | **Coefficient** | ***P*-value** | **Coefficient** | ***P*-value** |
| HIV VL at DEXA | <500 | 140 | - | - | - | - |
|  | >500 | 30 | -0.0205 | 0.094^◊^ | -0.1790 | 0.205 |
| Absolute CD4 count at DEXA |  | 168 | -0.0003 | 0.002 | -0.0003 | 0.000* |
| Maximum WHO clinical stage | 0-2 | 31 | - | - | - | - |
|  | 3 | 40 | 0.3176 | 0.177^◊^ | -0.1149 | 0.633 |
|  | 4 | 82 | 0.0522 | 0.800 | -0.1704 | 0.384 |
| *Treatment* |  |  |  |  |  |  |
| Abacavir |  | 106 | 0.1033 | 0.235 | - | - |
| Stavudine |  | 119 | 0.1158 | 0.469 | - | - |
| Tenofovir |  | 22 | 0.2746 | 0.044^◊^ | 0.2495 | 0.099 |
| Zidovudine |  | 73 | -0.1742 | 0.171^◊^ | -0.3331 | 0.019* |
| Lopinavir |  | 94 | 0.0470 | 0.696 | - | - |
| Nelfinavir |  | 44 | -0.1357 | 0.417 | - | - |
| Efavirenz |  | 104 | 0.0611 | 0.485 | - | - |
| *Duration Treatment* |  |  |  |  |  |  |
| HIV VL at DEXA scan | <500 | 140 | - | - | - | - |
|  | >500 | 30 | -0.0205 | 0.094^◊^ | -0.0790 | 0.570 |
| Absolute CD4^+^ T-cell count at DEXA |  | 168 | -0.0003 | 0.002^◊^ | -0.0003 | 0.002* |
| Maximum WHO clinical stage | 0-2 | 31 | - | - | - | - |
|  | 3 | 40 | 0.3176 | 0.177^◊^ | -0.0322 | 0.895 |
|  | 4 | 82 | 0.0522 | 0.800 | -0.1484 | 0.454 |
| Tenofovir |  | 22 | 0.1288 | 0.002^◊^ | 0.1131 | 0.026* |
| Zidovudine |  | 73 | 0.0172 | 0.069^◊^ | 0.0077 | 0.767 |

HIV VL= HIV viral load. DEXA= Dual Energy X-ray Absorptiometry. WHO= World Health Organisation. Multivariable analyses are adjusted for gender and country of origin. Lamivudine was used in all children and was therefore not included in the models.
^◊^=P<0.2 in univariable analysis.
*=P<0.05 after multivariable analysis.
